# Effects of additional context information in prescription drug information sheets on comprehension and risk and efficacy perceptions

**DOI:** 10.1186/s40545-021-00386-9

**Published:** 2022-03-01

**Authors:** Bridget Kelly, Amie O’Donoghue, Sarah Parvanta, Vanessa Boudewyns, Oluwamurewa Oguntimein, Carla Bann, Sue West, Janice Tzeng, Caroline Chandler, Gabriel Madson, Lauren McCormack

**Affiliations:** 1 RTI International, 701 13th Street NW, Ste. 750, Washington, DC 20005 USA; 2 Center for Drug Evaluation and Research, Food and Drug Administration, Silver Spring, USA

**Keywords:** Patient medication information, Context, Risk comprehension

## Abstract

**Objective:**

To determine how additional explanatory text (context) about drug side effects in a patient medication information handout affected comprehension and perceptions of risk and efficacy.

**Methods:**

We conducted an online experiment with a national sample of 1,119 U.S. adults with rheumatoid arthritis and related conditions, sampled through random-digit dialing, address-based sampling, and online ads. We randomized participants to receive one of several versions of a patient information handout for a fictitious drug, either with or without additional context, then measured comprehension and other outcomes.

**Results:**

Additional qualitative context about warnings and side effects resulted in lower comprehension of side effect information, but not information about uses of the drug or warnings. The effect of additional context on risk perceptions depended on whether the medication handout was delivered online or through the mail. Those who received a hardcopy of the handout with additional context had higher perceived risk of side effects than those who saw the version without additional context.

**Conclusion:**

More clarifying information is not always better and may lead to cognitive overload, inhibiting comprehension.

**Practice implications:**

Additional research should further explore effects of context in online vs. hard-copy formats before practice implications can be determined.

**Supplementary Information:**

The online version contains supplementary material available at 10.1186/s40545-021-00386-9.

## Introduction and background

Americans currently take more prescription drugs than ever before, with 55 percent taking at least one drug. Those taking prescription drugs use on average four different medications [1]. Patients report learning about their prescribed drugs from the pharmacy materials that accompany them [2, 3]. Given this, it is critical that the pharmacy materials the patients receive have clear and understandable information about the uses and risks of the medications they take.

To ensure that consumers have the information necessary to make informed decisions about whether to use prescription drugs and how to do so appropriately, the FDA has mandated use of Medication Guides [4–6] for certain drugs that pose “a serious and significant public health risk.” [4] Medication Guides (MedGuides) are developed by the pharmaceutical company that manufactures the drug, but must be approved by the FDA. These handouts contain information about uses and risks so that patients can make informed decisions on whether and how to use the drug and how to minimize the risk of adverse drug events. In addition to MedGuides and PPIs, most pharmacies distribute patient information with all prescriptions dispersed [7], but their formats vary dramatically. Identifying parameters that improve patients’ use of this patient medication information (PMI) involves examining the effects of variations of information presentation.

In order for PMI to be most useful for patients, best practices need to be developed for enhancing the comprehension of presented information. In line with learning theory suggested by Pan [8], adding contextual information may help readers remember information. This theory has been applied in studies on education, specific to helping students learn vocabulary [9]. In one experimental study of prescription drug information, researchers found that those who were provided with more information on drug risks were better able to understand drug side effects than those not given this contextual information [10].

However, some other studies on this topic have had mixed results [11]. In fact, some studies have concluded that adding more information can be detrimental to comprehension performance [12, 13]. In an experimental study of cognition, Engelhardt et al. [14] found that overdescribing an object, such as a square was associated with processing impairments. Some findings in the health and medical contexts also show that providing readers with additional information can result in cognitive overload or inhibit comprehension or recall [15]. Freer and colleagues [16] found that parents with a child in the neonatal intensive care unit participating in a research study had better understanding of study procedures when provided with simplified text than those who received a more detailed leaflet. Given these contradictory findings from previous literature and the limited literature on this topic in general, the purpose of this analysis is to examine the following research question: Does addition of qualitative contextual information about the risks of a drug enhance or lessen comprehension and ability to apply information? Specifically, we propose the following hypotheses:

H1: Participants who review PMI handouts with and without additional qualitative contextual information about drug warnings and side effects will demonstrate different levels of comprehension of drug risk information and application ability.

Little is known about the impact of including contextual information on patients’ perceptions of drug risk and benefit. Rothman and Kiviniemi [17] theorized that people will consider the antecedents and consequences of their health problems when provided with contextualized information, which may change their health risk perceptions. Keown and colleagues [10] also found that those who received more detailed descriptions of drug risks rated the seriousness of the risks as lower than those who did not receive the additional information on drug risks.

Although Keown and colleagues found that adding contextual information on drug risk reduced patient’s perceived risk, other research suggests that the addition of such information could have the opposite effect. According to risk communication research, perceived threat heightens when a health risk appears serious and likely to happen [18]. Thus, messages containing additional context about the severity of a drug’s risks may magnify the severity, and subsequently lead to greater risk perceptions. As such, we predict the following:

H2: Those who review PMI handouts with additional qualitative contextual information about drug warnings and side effects will perceive greater drug safety risks related to the drug.

Previous research on direct-to-consumer advertising suggests that risk and efficacy perceptions have an inverse relationship, i.e., increasing a person’s perceptions of drug efficacy reduces perceptions of risk [19–24]. Consequently, if adding contextual information about a drug’s risk increases risk perceptions, it may also reduce the perceived effectiveness of the drug.

H3: Those who review PMI handouts with additional qualitative contextual information about drug warnings and side effects will perceive lower efficacy related to the drug.

Various content and formatting decisions are often tested in isolation. For example, some previous research examined the readability level of patient information distributed with numerous types of prescription medications [25–27], whereas other research explored strategies such as grouping text together in chunks to improve its organization. Whether certain strategies interact to either improve or inhibit patient retention and comprehension is unclear. In previous analyses from this study, we found that two alternate one-page formats improved comprehension compared to a more standard medication guide [28]. However, those analyses did not examine the impact of context in the alternate formats, nor did they look for any interaction effects between context and format. Therefore, in addition to exploring the direct effect of contextual information on patient comprehension and risk and efficacy perceptions, this analysis will also explore whether various formats or varied modes of information delivery—print vs. online—moderate the effect of context on the key outcomes.

H4: Format will moderate the effect of context on key outcomes.

H5: Mode of delivery will moderate the effect of context on key outcomes.

The goal of this analysis is to experimentally test patient medication handouts that have been strategically enhanced to improve patient comprehension and understanding of risks and perceptions of efficacy. Specifically, this study focuses on the impact of adding contextual information to explain the side effects and warnings for the drug and explores whether such information is moderated by various formatting styles and modes of delivery. The results of this study will provide FDA with information about the usefulness of alternate formats for patient information handouts.

## Methods

The stimulus for this study was designed around a fictitious drug, “Rheutopia,” which was modeled after an existing injectable indicated for the treatment of rheumatoid arthritis, ankylosing spondylitis and plaque psoriasis. Rheumatoid arthritis impacts approximately 2% of the U.S. adult population [29]. It is approximately twice as common in women as men and affects some groups disproportionately, such as Pima Native Americans, in which rates are as much as 10 times higher than for other groups [30, 31]. Plaque psoriasis and ankylosing spondylitis are less prevalent but can be treated with the same medications, and thus were included in the study. The procedures for this study have been described in two earlier publications [28, 32]. Briefly, we randomized participants to receive an information sheet either online or on hardcopy through the mail and then used an online survey to interview patients who were 18 years of age or older with self-reported rheumatoid arthritis, ankylosing spondylitis, or plaque psoriasis identified via GfK Custom Research’s KnowledgePanel and a partner panel to test five different versions of a handout for a fictitious drug. Two of the five versions had additional context information. Specifically, the “context versions” provided additional explanatory information about the drug’s side effects and warnings, whereas the no context version did not include the explanatory information. The context information we added was limited to qualitative descriptions about why it was important to look for specific symptoms or to tell your doctor about certain side effects. For example, the no-context version included a statement, “Call your doctor right away if you develop chills, swollen lymph nodes, night sweats, fever or weight loss.” The context version included the additional sentence to explain why these symptoms were important to note: “You may have a higher chance of getting lymph node cancer.” Additional context did not include any incidence rates or risk likelihood information. A fifth group, the control group, received a 4-page Medication guide. Since that version was not relevant for the context comparison, the control group is excluded from the analyses described here. The study handouts can be found in Additional file 1: Appendix S1 with additional context information highlighted in yellow.

In addition, all handouts included one of two format variations: Bubbles or over-the-counter (OTC). The Bubbles version has text formatted into rounded boxes (i.e., bubbles) and organized as two vertical columns on the page. The OTC format has information organized into boxes that run the full width of the page, analogous to the OTC label format approved by FDA and currently in use (nonprescription drug product labeling requirement, 1999). Thus, we used four versions of the handout in this analysis: (1) Context Bubbles, (2) Context OTC, (3) No Context Bubbles, and (4) No Context OTC. A visual depiction of the study design has been published elsewhere [28].

### Measures

After reading the handouts, participants responded to the online survey questions about their self-reported preferences (e.g., ease of understanding, clarity of information), their confidence in the ability to understand the information in the handout, and their perceived risk and efficacy of the fictitious drug for others. Participants also answered objective measures of comprehension and application. The comprehension measure was a composite of three subscales: (a) side effects and risks; (b) benefits and uses; and (c) topics to discuss with a doctor before taking Rheutopia. The survey had both true/false questions (e.g., “According to the patient information sheet, people who take Rheutopia can develop dry skin”) and multiple-response questions (e.g., “Please check all the possible Rheutopia side effects mentioned in the handout”). We coded responses to the closed-ended questions as correct or incorrect and developed subscale scores as a percentage of correct items. We computed an overall composite comprehension score by weighting the three subscales based on their factor loadings; possible scores ranged from 0 to 100.

We measured application (respondents’ ability to apply the information they read) with three closed-ended items that presented different scenarios (e.g., “Jack missed his dose of Rheutopia. According to the patient information sheet, what should Jack do?”). Responses were coded as either incorrect or correct; the application score is the percentage of correct answers.

Subjective health literacy was measured using an average of 3 items from the European subjective health literacy scale; responses ranged from 1 [very difficult] to 4 [very easy]). The three specific items in this measure are detailed in Table 1 with the remaining measures.

**Table 1:** Key measures

Construct	Measure	Answer options
Comprehension	The comprehension measures reflected information retrieval, primarily whether participants could locate and understand the information in the handouts. We assessed three issues related to the information in the handout on the drug: (a) side effects; (b) uses; and (c) warnings/topics to discuss with a doctor before taking Rheutopia. and developed subscale scores by counting the number of correct items for each of the side effects, uses and warnings question subsets	We included both true/false questions (e.g., “According to the patient information sheet, people who take Rheutopia can develop dry skin”) and multiple-response questions (e.g., “Please check all the possible Rheutopia side effects mentioned in the handout”). We coded responses as correct or incorrect. The comprehension score is the % of correct answers
Application	Application questions reflected the participants’ ability to use the information in the handouts to respond to three hypothetical scenarios (e.g., “Jack missed his dose of Rheutopia. According to the patient information sheet, what should Jack do?”)	We coded responses as either correct or incorrect; the application score is the percentage of correct answers
Perceived clarity	The information about the risks of Rheutopia in the patient information sheet is… The information about the uses of Rheutopia in the patient information sheet is…	1 = not at all clear 5 = very clear (two items were averaged)
Perceived ease of understanding	The information about the risks of Rheutopia in the patient information sheet is… The information about the uses of Rheutopia in the patient information sheet is…	1 = easy to understand, 5 = hard to understand (reverse coded; two items averaged)
Comprehension confidence	How confident are you that you can understand the information on this sheet?	1 = not at all confident, 5 = very confident
Risk likelihood	If 100 people take Rheutopia, how many people will have any side effects?	Response options ranged from 0 to 100
Efficacy likelihood	… [for] how many people will Rheutopia improve symptoms of RA?	Response options ranged from 0 to 100
Perceived risk magnitude	If Rheutopia did cause a person to have side effects, how serious would they be?	1 = not serious at all, 5 = very serious
Efficacy magnitude	How much do you think Rheutopia is going to help the average person’s Rheumatoid arthritis?	1 = would help RA very little, 5 = would help RA a lot)
Subjective health literacy (average of 3 items from the European health literacy scale)	On a scale from very easy to very difficult, how easy would you say it is to: understand your doctor’s or pharmacist’s instruction on how to take a prescribed medicine?; follow instructions from your doctor or pharmacist?; understand the information that comes with your medicine?	1 = very easy, 4 = very difficult 5 = don’t know. (reverse coded) Mean = 3.42 (SD = 0.56), out of 4
Perceived illness knowledge	In general, how much would you say you know about [CONDITION]? Would you say you know:	1 = nothing at all, 5 = a lot. The mean illness knowledge score was 3.63 (SD = 0.98) out of 5

### Statistical analysis

We conducted univariate and bivariate analyses (*t* tests) followed by linear regression to evaluate the effects of additional context on key outcomes. We examined correlations of all outcome measures to understand whether self-reported measures of clarity of the information and ease of understanding were well correlated with more objective measures of comprehension and application. To ensure all variables were comparable in regression models, we standardized all scales to have a mean 0 and standard deviation of 1. We used linear regression models to explore differences in outcomes by handout version (context vs. no context and Bubbles vs. OTC) after controlling for other possible predictors, including age, gender, race, ethnicity, education level, subjective health literacy, perceived illness knowledge, time since diagnosis and mode of handout administration.^1^ Finally, to understand if the impact of context within the patient medication information handout depended on handout mode, format, or other participant characteristics, we tested for two-way interactions between context and format, context and mode, as well as context and the other variables. We also tested for a three-way interaction between mode, format, and context. Following typical procedures for tests of interactions, any non-significant interactions were dropped from the models. All analyses were conducted using SAS software version 9.3 [33].

We conducted the study between November 2012 and January 2013, with all study procedures approved by the Institutional Review Boards at both institutions.

## Results and discussion

### Results

A total of 1397 (58%) of the 2394 panelists invited to participate completed the study. We report participant characteristics in Table 2. Sixty-five percent had rheumatoid arthritis, 25% had plaque psoriasis, 6% had ankylosing spondylitis, and approximately 5% had two or more of the target medical conditions. Participant characteristics were similar across study groups, with the majority being Non-Hispanic White and female. The higher percentage of females in the study likely reflects the higher prevalence of RA in women in the population [34, 35].

**Table 2:** Demographic characteristics of study participants

Characteristic	*n* (%)
Sex	
Female	735 (66%)
Male	384 (34%)
Age range (years)	
18–49	345 (31%)
50–59	338 (30%)
60–69	282 (25%)
70 or older	154 (14%)
Race/Ethnicity	
White or Caucasian	876 (78%)
Black or African American	96 (9%)
Hispanic	80 (7%)
Other	67 (6%)
Education	
High school or less	266 (24%)
Some college	446 (40%)
College graduate	407 (36%)
Medical condition	
Rheumatoid arthritis	727 (65%)
Ankylosing spondylitis	60 (5%)
Plaque psoriasis	274 (24%)
2 or more medical conditions	58 (5%)
Subjective health literacy…mean ± SD^a^	3.42 ± 0.56
Illness knowledge…mean ± SD^b^	3.63 ± 0.98

*N* = 1119

^a^Scale ranged from 1 (*very difficult*) to 4 (*very easy*)

^b^One item: responses ranged from 1 (*know nothing at all*) to 5 (*know a lot*)

Table 3 shows the correlations among the outcome variables and descriptive scores for all key outcomes. Overall, participants were confident that they could understand the content and they agreed that the information was clear. Comprehension measures were strongly correlated with each other and with application. In general, although comprehension confidence and actual comprehension measures were significantly correlated, these correlations were not particularly high (*r* < 0.30, *P* < 0.001). Correlations between the risk and efficacy variables were small, but in the expected negative direction (see Table 3).

**Table 3:** Correlations and descriptive statistics for key outcomes (*N* = 1119)

Variables	1	2	3	4	5	6	7	8	9	10	11
Correlations											
1.Perceived clarity	1.00										
2.Ease of understanding	0.43***	1.00									
3.Comprehension confidence	0.40***	0.22***	1.00								
4.Comprehension-uses	0.27***	0.21***	0.29***	1.00							
5.Comprehension-risks	0.26***	0.23***	0.27***	0.54***	1.00						
6.Comprehension-warnings	0.24***	0.17***	0.27***	0.56***	0.52***	1.00					
7.Application	0.27***	0.18***	0.27***	0.52***	0.46***	0.47***	1.00				
8.Risk Likelihood	0.02	− 0.01	− 0.06	− 0.02	0.01	0.02	0.03	1.00			
9.Efficacy Likelihood	0.19***	0.03	0.16***	0.07*	0.08*	0.04	0.05	− 0.11***	1.00		
10.Risk Magnitude	0.05	0.02	− 0.03	− 0.10**	− 0.03	− 0.09**	− 0.03	0.25***	− 0.17***	1.00	
11.Efficacy Magnitude	0.28***	0.09***	0.24***	0.03	0.04	0.01	0.01	− 0.15***	0.60***	− 0.11***	1.00
	**Perceived clarity**	**Ease of under-standing**	**Comp. Confid**	**Comp.-Uses**	**Comp- Risks**	**Comp-Contra**	**App**	**Risk likely hood**	**Effic. likely hood**	**Risk Mgt**	**Effic. Mgt**

All bold correlations are significantly different from 0, *p < .05 **p < .01, *p* < .001***. Due to some missing data, some correlations are based on 1115–1,119 participants

#### Self-reported understanding and comprehension confidence

Results from regression models, controlling for patient characteristics and exploring two- and three-way interactions found no significant effects of format or context on perceived clarity, ease of understanding or comprehension confidence (data not shown in tables). Some participant characteristics did directly affect perceived clarity, ease of understanding and comprehension confidence. Specifically, those who were age 50–59 (*B* = 0.16; *P* =  < 0.001) and 60–69 (*B* = 0.10; *P* =  < 0.01) (compared to those younger than 50) were more likely to have higher ease of understanding. The same was true for those with higher health literacy (*B* = 0.29; *P* =  < 0.001). Those with higher health literacy (*B* = 0.43; *P* =  < 0.001) and higher illness knowledge (*B* = 0.09; *P* =  < 0.01) were also more likely to say the information was clear.

Those with some college (*B* = 0.12; *P* =  < 0.01) or a college degree or more (*B* = 0.17; *P* =  < 0.001) those with higher health literacy (*B* = 0.33; *P* =  < 0.001) and with greater illness knowledge (*B* = 0.14; *P* < 0.001) had higher comprehension confidence. Age, race and gender were not associated with comprehension confidence.

#### Comprehension of information

##### Side effects

**Fig. 1:**
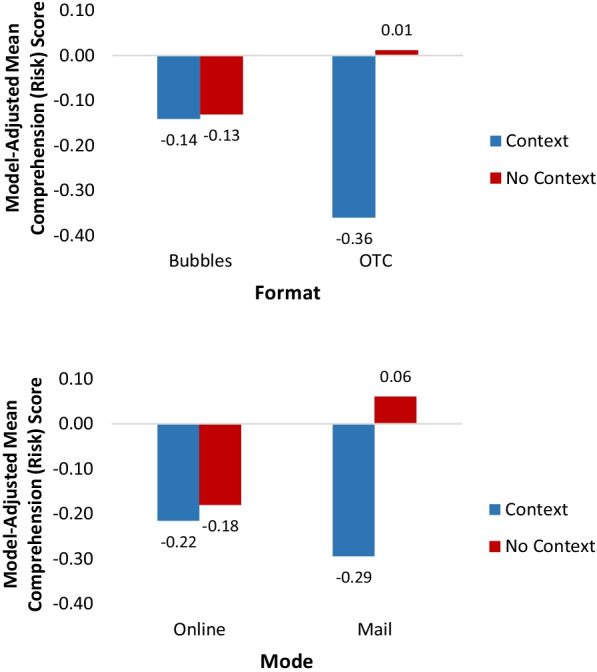
Comprehension of Risks by Format and Context and by Mode and Context. For comprehension of risks (top graph), no context resulted in significantly more comprehension in the OTC format, but not the Bubbles format. Mode also mattered for comprehension of risks (bottom graph), such that in the mailed version, additional context resulted in lower comprehension, while the same was not true for the online version

We observed a significant main effect of context on comprehension of side effects (see Table 4). Format and mode moderated this effect. We examined the effect of format x context first, then mode x context. The format by context interaction revealed that although there was no effect of context on comprehension of side effects for those who saw the Bubbles version, compared with no context, context resulted in lower comprehension of side effects for those who saw the OTC version (Fig. 1).

**Table 4:** Regression models predicting comprehension and application

Characteristic	Comprehension outcome variables			
	Risks/side effects	Uses	Warnings	Application
	*B* (95% CI)	*B* (95% CI)	*B* (95% CI)	*B* (95% CI)
Context				
Present	− 0.54*** (− 0.74, − 0.34)	− 0.06 (− 0.17, 0.05)	− 0.10 (− 0.21, 0.02)	− 0.07 (− 0.18, 0.04)
Absent	REF	REF	REF	REF
Format				
Bubbles	− 0.14 (− 0.31, 0.04)	0.06 (− 0.05, 0.17)	0.01 (− 0.10, 0.13)	0.13* (0.02, 0.24)
OTC	REF	REF	REF	REF
Mode				
Online (Electronic handout)	− 0.23** (− 0.41, − 0.06)	− 0.09 (− 0.21, 0.02)	− 0.34*** (− 0.46, − 0.22)	0.09 (− 0.03. 0.20)
Mail (Print handout)	REF	REF	REF	REF
Context × Mode	0.32** (0.09, 0.54)	NA	NA	NA
Context x Format	0.36** (0.14, 0.58)	NA	NA	NA
Gender				
Male	− 0.17** (− 0.29, − 0.05)	− 0.33*** (− 0.45, − 0.20)	− 0.18** (− 0.30, − 0.05)	− 0.19** (− 0.32. − 0.07)
Female	REF	REF	REF	REF
Age				
< 50	REF	REF	REF	REF
50–59	0.18* (0.03, 0.33)	0.06 (− 0.09, 0.21)	0.05 (− 0.11, 0.20)	0.02 (− 0.12. 0.16)
60–69	0.17* (0.02, 0.32)	0.10 (− 0.05, 0.24)	0.12 (− 0.03, 0.28)	0.14 (− 0.01, 0.28)
70 +	− 0.08 (− 0.26, 0.11)	− 0.01 (− 0.19, 0.18)	− 0.05 (− 0.23, 0.14)	− 0.05 (− 0.28, 0.19)
Education				
High school or less	REF	REF	REF	REF
Some college	− 0.05 (− 0.20, 0.09)	0.04 (− 0.11, 0.19)	0.13 (− 0.02, 0.28)	0.04 (− 0.11, 0.19)
College or more	− 0.05 (− 0.20, 0.10)	0.22** (0.07, 0.37)	0.23** (0.07, 0.38)	0.10 (− 0.05, 0.25)
Race/ethnicity				
Non-Hispanic White	REF	REF	REF	REF
Non-Hispanic Black	− 0.08 (− 0.30, 0.14)	− 0.38** (− 0.58, − 0.17)	− 0.33** (− 0.55, − 0.12)	− 0.16 (− 0.38, 0.05)
Hispanic	− 0.17 (− 0.39, 0.04)	− 0.18 (− 0.41, 0.05)	− 0.26* (− 0.50, − 0.02)	− 0.08 (− 0.32. 0.16)
Other	− 0.04 (− 0.26, 0.19)	− 0.24 (− 0.48, 0.01)	− 0.25* (− 0.48, − 0.02)	− 0.05 (− 0.28, 0.19)
Time since diagnosis				
< 6 months	− 0.22 (− 0.49, 0.05)	− 0.30* (− 0.58, − 0.02)	− 0.26 (− 0.54, 0.01)	− 0.39** (− 0.68, − 0.09)
6–12 months	− 0.48*** (− 0.70, − 0.25)	− 0.70** (− 0.98, − 0.41)	− 0.34** (− 0.57, − 0.10)	− 0.36** (− 0.61, − 0.11)
1–5 years	− 0.18* (− 0.32, − 0.04)	− 0.15* (− 0.28, − 0.02)	− 0.09 (− 0.23, 0.05)	− 0.11 (− 0.24, 0.02)
> 5 years	REF	REF	REF	REF
Subjective health literacy	0.19** (0.08, 0.30)	0.22** (0.11, 0.34)	0.19** (0.07, 0.31)	0.29*** (0.17, 0.42)
Illness knowledge	0.05 (− 0.02, 0.11)	0.01 (− 0.05, 0.07)	− 0.03 (− 0.09, 0.04)	0.04 (− 0.02, 0.10)

^*^p value < .05; **p value < .01; ***p value < .001

*NA* Interaction did not significantly predict outcome variable, so we report the model that did not include this interaction

Examining the mode by context interaction revealed that the effect of context on comprehension of side effects was only found for the mailed version of the handout. Specifically, those who viewed the mailed version of the handout with context had reduced comprehension of side effects (Fig. 1) compared to those who viewed the mailed version with no context. By contrast, there was no difference in mean comprehension of side effects when comparing the context to the no context versions for those who viewed the stimuli online. Demographic predictors included gender, age and health literacy. Females, those ages 50–69, and those with higher health literacy had higher comprehension of side effects (data not shown).

##### Comprehension of uses and warnings

For comprehension about the uses of the drug, there were no main effects of context or format and no interactions. For comprehension about the warnings of the drug, there were also no main effects of context or format and no interactions. Differences for demographic groups are found in Table 4.

#### Application of the information

Whereas context was not related to application, those who received the Bubbles format were better able to apply the information than those who saw the OTC version. Differences by demographic group are found in Table 4.

#### Risk and efficacy likelihood and magnitude

Multiple regression analyses indicated that the effects of context on drug risk and efficacy perceptions were marginal, with only one main effect (on efficacy likelihood) (see Table 5).

**Table 5:** Regression models predicting risk likelihood, efficacy likelihood, risk magnitude and efficacy magnitude

Characteristic	Outcome variables			
	Risk likelihood	Efficacy likelihood	Risk magnitude	Efficacy magnitude
	*B* (95% CI)	*B* (95% CI)	*B* (95% CI)	*B* (95% CI)
Context				
Present	0.11 (− 0.10, 0.33)	− 0.12* (− 0.24, 0.00)	0.04 (− 0.08, 0.16)	0.05 (− 0.07, 0.16)
Absent	REF	REF	REF	REF
Format				
Bubbles	0.01 (− 0.16, 0.19)	− 0.09 (− 0.20, 0.03)	− 0.05 (− 0.17, 0.07)	− 0.02 (− 0.14, 0.09)
OTC	REF	REF	REF	REF
Mode				
Online (Electronic handout)	0.09 (− 0.09, 0.26)	− 0.06 (− 0.17, 0.06)	0.03 (− 0.09, 0.15)	0.00 (− 0.12. 0.12)
Mail (Print handout)	REF	REF	REF	REF
Context × Mode	− 0.22 (− 0.46, 0.02)	NA	NA	NA
Context × Format	0.05 (− 0.20, 0.29)	NA	NA	NA
Gender				
Male	− 0.13 (− 0.26, 0.00)	0.09 (− 0.04, 0.21)	− 0.02 (− 0.15, 0.10)	0.11 (− 0.02. 0.24)
Female	REF	REF	REF	REF
Age				
< 50	REF	REF	REF	REF
50–59	0.12 (− 0.03, 0.27)	− 0.14 (− 0.28, 0.01)	0.20* (0.05, 0.36)	− 0.07 (− 0.21. 0.07)
60–69	0.08 (− 0.08, 0.24)	− 0.34*** (− 0.50, − 0.19)	0.17* (0.01, 0.33)	− 0.29** (− 0.44, − 0.13)
70 +	0.05 (− 0.15, 0.25)	− 0.43*** (− 0.63, − 0.22)	0.30** (0.10, 0.50)	− 0.30** (− 0.50, 0.10)
Education				
High school or less	REF	REF	REF	REF
Some college	− 0.03 (− 0.18, 0.13)	0.06 (− 0.10, 0.21)	− 0.13 (− 0.29, 0.02)	0.06 (− 0.09, 0.21)
College or more	− 0.06 (− 0.22, 0.10)	0.14 (− 0.02, 0.30)	− 0.27** (− 0.43, 0.11)	0.12 (− 0.03, 0.28)
Race/Ethnicity				
Non-Hispanic White	REF	REF	REF	REF
Non-Hispanic Black	0.21 (− 0.01, 0.43)	− 0.03 (− 0.24, 0.19)	0.32** (0.09, 0.56)	0.05 (− 0.18, 0.28)
Hispanic	0.24* (0.01, 0.48)	− 0.03 (− 0.24, 0.19)	0.35** (0.13, 0.57)	0.14 (− 0.08. 0.36)
Other	− 0.02 (− 0.26, 0.23)	− 0.03 (− 0.25, 0.18)	0.18 (− 0.08, 0.45)	0.10 (− 0.15, 0.35)
Time since diagnosis				
< 6 months	0.39* (0.08, 0.69)	0.19 (− 0.08, 0.46)	0.26 (− 0.01, 0.53)	0.05 (− 0.21, 0.32)
6–12 months	0.25* (0.01, 0.49)	0.07 (− 0.12, 0.26)	0.14 (− 0.07, 0.35)	0.09 (− 0.10, 0.28)
1–5 years	− 0.05 (− 0.19, 0.08)	0.09 (− 0.05, 0.23)	− 0.08 (− 0.22, 0.06)	0.15* (0.01, 0.29)
> 5 years	REF	REF	REF	REF
Subjective health literacy	− 0.02 (− 0.13, 0.10)	0.19** (0.07, 0.31)	− 0.07 (− 0.19, 0.04)	0.32*** (0.20, 0.43)
Illness knowledge	− 0.03 (− 0.09, 0.04)	0.07* (0.01, 0.14)	− 0.02 (− 0.09, 0.05)	0.09** (0.03, 0.16)

^*^p value < .05; **p value < .01; ***p value < .001

*NA* Interaction did not significantly predict outcome variable, so we report the model that did not include this interaction

Perceived efficacy likelihood decreased when additional context was present. Context did not significantly influence perceived risk magnitude or efficacy magnitude. In addition, mode and format did not moderate the impact of context on perceived efficacy likelihood, risk magnitude or efficacy magnitude.

## Discussion

The aim of this study was to explore the effect of providing additional qualitative context information about side effects and warnings on key outcomes related to patients’ comprehension, application of medical information, and risk and efficacy perceptions. The findings present a somewhat complex picture of these effects.

We hypothesized (H1) that the presence or absence of additional context for some risk information would influence participants’ comprehension and application of the risk information. Competing lines of research suggested that context would either aid [10] or hinder [15] the comprehension of risk information. Our study supported the latter, finding that additional qualitative context about why certain side effects were important *reduced* comprehension. While this effect was only statistically significant for comprehension of side effect information, the coefficients for all other comprehension measures were in the same direction. It makes sense that there would be no difference for comprehension of uses of the drug, as there was no additional context in the sections of the handout about uses. There was some added context in the section about warnings, but this did not result in statistically significant effects on comprehension of warnings.

It is clear from the interaction results that the additional context mattered more for the OTC version than for the Bubbles version. That is, participants in the Bubbles condition showed no comprehension decrement, whereas those viewing the OTC version were more negatively affected by the additional context. It is unclear what makes the Bubbles format more forgiving of extra context. Perhaps participants are accustomed to the limited information in current OTC labels, thus the additional context was jarring. Or it may be that the formatting of the Bubbles condition, which clearly separated content into sections, helped to make the additional information clearer and easier to digest.

The results also suggest that mode of delivery mattered in determining effects of additional context: There was a difference in comprehension for those who viewed the mailed version of the handout with context vs. no context, but the same was not true for the online version. This result deserves some further exploration in future research.

The addition of context did not affect comprehension confidence, perceived ease of understanding, or perceived clarity of the information. This is inconsistent with Keown [10], who found that participants who received additional context reported the risks easier to understand. However, the risk information provided there was quantitative and specific—detailing the frequency with which risks were likely to occur. The additional information in our context condition included somewhat tangential and distinctly qualitative information. Our findings suggest that qualitative information may be perceived as noise, whereas other research shows that quantitative information may be useful [24].

Overall, then, it appears that additional qualitative context for side effects and warnings in PMI does not help participants find and understand the information they read and may in some cases inhibit this effort. In no cases did we find an advantage in terms of comprehension or application for additional context of risk. This has implications for the development of these informational documents and supports previous research suggesting that informational documents maintain as little information as possible while maintaining the important message of the document [15, 16].

In addition to findings regarding context alone and in combination with format, we observed an interaction between context and mode of administration which showed that people who received the PMI in the mail demonstrated a comprehension decrement with additional context, but those viewing it online did not. Evidence from other research supports a finding that the mailed patient information handout might have a greater impact than a handout delivered online. In a previous analysis of these handouts, mode directly influenced comprehension of the handout information; participants receiving the mailed version had higher comprehension than those receiving the online version [28]. It could be that receiving the handout in advance through the mail made it seem official or important in a way that was not conveyed through the online version. Or it could be that the medium itself, print vs. online, provided different opportunities for the contextual information to come across. Our findings are consistent with Sundar and Narayan [36], who found an advantage of memory for print vs. online information. However, more recent studies have shown that the modality of the information plays little role, at least concerning news information [37, 38]. Suri et al. [39] found differences between print and online coupons depending on the involvement level of participants. Since we did not measure involvement, we cannot assess whether it played a role in our findings. However, our findings match Suri et al.’s low involvement condition. Although we limited our sample to individuals who had been diagnosed with the medical condition the drug treats, it is likely that participants did not expect to gain any valuable information from a survey, making this a situation that did not mimic an actual treatment decision. Future research utilizing samples at the physician’s office or the pharmacy would be useful to gauge responses in a more realistic setting.

The results for the efficacy likelihood outcome are complex. In the unadjusted model, there was no significant effect of context on efficacy likelihood. However, when we controlled for demographics and other variables, the negative coefficient became more negative and statistically significant. One possible explanation is that the significant negative effect of additional context on efficacy likelihood is suppressed by health literacy. The additional context may result in lower efficacy likelihood, because the additional information about risks somehow affects perceptions of how well the drug works, which might be the case for people with lower health literacy who are more overwhelmed or overloaded by the additional context. To test this possible explanation, we ran the adjusted model with all other control variables except health literacy. The coefficient for context was not significant (data not shown), lending support to the theory that health literacy may be suppressing the effect of context on efficacy likelihood. This possible explanation requires further exploration in future research.

Another important finding related to health literacy was that those with higher health literacy, as well as higher education, had greater confidence in their own ability to understand the information. This is important, because confidence in one’s ability to understand the information may be an important predictor of actual comprehension. Research on informed consent has found that self-efficacy about comprehension can be a mediator of health literacy’s effects on outcomes, such as confusion and feelings of being well informed [40]. Consideration of this need to build confidence may be an important avenue for intervention in efforts to improve patient comprehension of prescription drug materials.

The study had some limitations that deserve mention. The additional “context” information in this case was limited to a few additional sentences in the section of the information sheet on side effects and warnings. This is an important point, as the *type* of context probably matters [41]. There are various other ways context can be defined and these may produce different effects than those found here. In the Keown study, context was defined as the common name of the side effect, whether it occurred intermittently or continuously while taking the drug and whether it had temporary or permanent consequences. Information about temporary or permanent consequences may have very different effects on risk perceptions than information clarifying for whom a particular risk is relevant.

An additional limitation may come from our decision to measure perceived risk and efficacy in terms of others rather than oneself. We observed a lack of significant findings related to risk and efficacy magnitude outcomes and small effects for risk and efficacy likelihood outcomes. If we instead measured these items in terms of perceived risk and efficacy to oneself, we may have found stronger results.

Finally, Likert scales are technically not continuous variables, but ordinal and there is debate in the literature regarding their use as continuous variables. However, many studies have shown that parametric statistics are robust to potential violation of regression assumptions [42].

## Conclusion

This study compared several versions of a patient information sheet to determine whether additional context explaining why certain side effects or warnings were important helped or hindered comprehension of risks. Findings provide some insight into the impact of qualitative context and format on patient comprehension and application of medication information as well as patient risk and efficacy perceptions. Despite participants in all groups feeling highly confident in their ability to understand the information and reporting that the information was clear and easy to understand, the addition of contextual information about side effects and warnings appeared to inhibit comprehension.

## Practical implications

The comparison of perceived ability to comprehend the information and objective comprehension measures underlines the importance of measuring comprehension objectively. Addition of context information affected risk perceptions for those who viewed the mailed handout and influenced beliefs about likely effectiveness. The findings suggest that more information may not be better when communicating about prescription drug uses and side effects. Keeping patient materials clear and simple is important in ensuring comprehension. These findings may have implications for informed consent and other materials distributed in healthcare settings. Further research is needed to identify appropriate measures of these constructs.

## Supplementary Information

**Additional file 1: Appendix S1.** Patient medication information handouts.

## Data Availability

The data sets used and/or analyzed during the current study are available from the corresponding author on reasonable request.

## Footnotes

1 Not all measures were normally distributed upon examination of histograms. Thus, we also ran the analyses using Poisson regression. The results were consistent with those from the OLS regression models.

## Declarations

### Confidentiality

I confirm all patient/personal identifiers have been removed or disguised so the patient/person (s) described are not identifiable and cannot be identified through the details of the story.
