# Supplementary material for: Effects of additional context information in prescription drug information sheets on comprehension and risk and efficacy perceptions
Source: J Pharm Policy Pract. 2022 Mar 1;15:15. doi: 10.1186/s40545-021-00386-9 (PMC8887124; doi:10.1186/s40545-021-00386-9)
Supplement: Supplementary file 1 — Additional file 1: Appendix S1. Patient medication information handouts. [file 40545_2021_386_MOESM1_ESM.docx]

Appendix 1. Patient Medication Information Handouts

| OTC with No Context | OTC with Context | Medication Guide  (page 1 of 4) |
| --- | --- | --- |
| 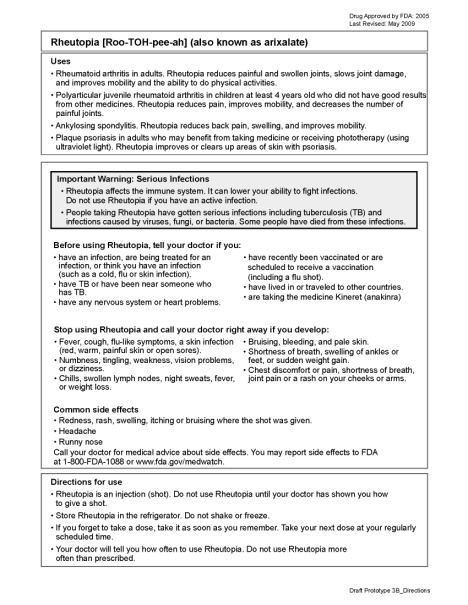 | 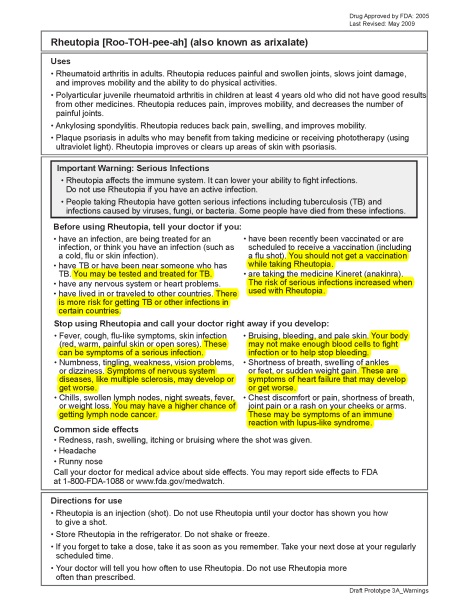 | 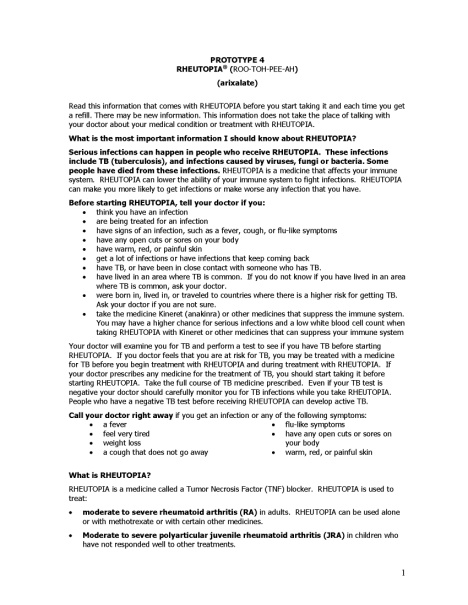 |
| Bubbles with No Context | Bubbles with Context |  |
| 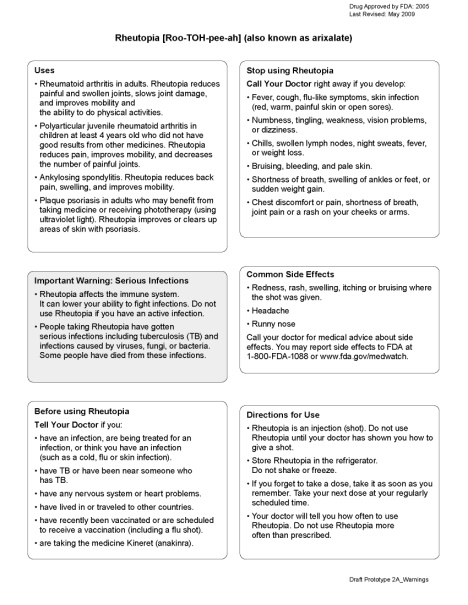 | 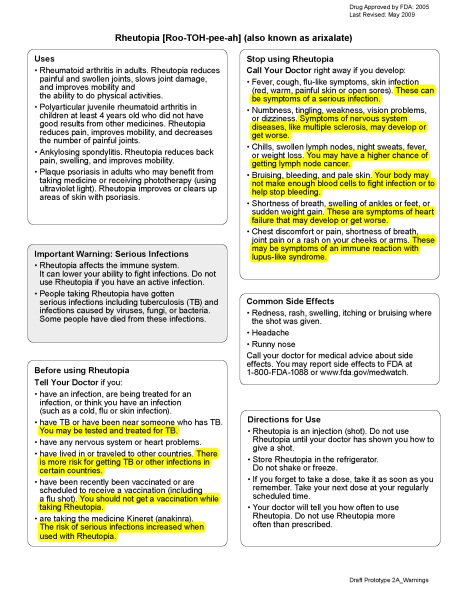 |  |
